# Esmolol Alleviates Sepsis‐Associated Myocardial Injury by Acting on the APAF1 and JAK2/STAT3 Inflammatory Pathways

**DOI:** 10.1155/cdr/2843754

**Published:** 2026-10-12

**Authors:** Lei Zhao, Hua Dong, Shimin Dong

**Affiliations:** ^1^ Department of Emergency Medicine Hebei Medical University Fourth Hospital Shijiazhuang Hebei China; ^2^ Department of Radiation Oncology Hebei Medical University Fourth Hospital Shijiazhuang Hebei China; ^3^ Department of Emergency Hebei Medical University Third Hospital Shijiazhuang Hebei China

**Keywords:** APAF1, apoptosis, esmolol, inflammation, sepsis-associated myocardial injury

## Abstract

**Objective:**

The aim of this study is to explore the protective effect of esmolol against sepsis‐associated myocardial injury (SAMI) and its underlying molecular mechanisms.

**Methods:**

SAMI‐related differentially expressed genes were identified from the Gene Expression Omnibus database (GSE267388). Potential targets of esmolol were predicted using PharmMapper. Overlapping genes were used for enrichment and molecular docking analyses. H9C2 cells were treated with LPS to establish an injury model, followed by esmolol treatment. Cell viability, apoptosis, oxidative stress, and mitochondrial function were also assessed. The expressions of apoptotic protease activating factor 1 (APAF1) and JAK2/STAT3 pathway molecules were detected using reverse transcription‐quantitative polymerase chain reaction (RT‐qPCR) and Western blotting. A rat model of sepsis was used to verify cardiac function, inflammation, oxidative stress, and related protein expression.

**Results:**

Six core targets were screened, and esmolol showed the strongest binding to APAF1. Esmolol increased cell viability; reduced apoptosis, ROS, and MDA; restored mitochondrial membrane potential; downregulated APAF1, Bax, cleaved‐caspase‐9/3; and suppressed JAK2/STAT3 phosphorylation. Esmolol reversed APAF1 overexpression‐induced injury. In vivo, esmolol alleviated myocardial pathological injury, decreased the concentrations of myocardial markers and inflammatory factors, and inhibited the APAF1 and JAK2/STAT3 pathways.

**Conclusion:**

Esmolol protects against SAMI by dual inhibition of the APAF1 mitochondrial apoptotic and JAK2/STAT3 inflammatory pathways, with APAF1 likely serving as a key downstream target of esmolol.

## 1. Introduction

Sepsis is a life‐threatening multiple organ dysfunction syndrome caused by systemic and dysregulated inflammatory responses to infections [1]. The heart is one of the most vulnerable organs to sepsis. Sepsis‐associated myocardial injury (SAMI) is a common and severe complication characterized by impaired cardiac systolic and diastolic function, increased cardiomyocyte apoptosis, and excessive release of proinflammatory cytokines. SAMI is an independent risk factor for poor prognosis and mortality of patients with sepsis, with a mortality rate exceeding 70% [2, 3]. Currently, specific targeted therapies for SAMI are lacking. Therefore, elucidating the underlying molecular mechanisms and identifying effective therapeutic targets are of great clinical and scientific significance.

Among the various therapeutic strategies explored for SAMI, *β*‐adrenergic receptor blockade has garnered increasing attention due to its ability to modulate the excessive sympathetic activation and inflammatory cascades characteristic of sepsis. Esmolol is a short‐acting and highly selective *β*1‐adrenergic receptor blocker that is widely used clinically for heart rate (HR) control in patients with cardiomyopathy and management of postoperative hypertension. A growing body of evidence suggests that esmolol exerts anti‐inflammatory and antiapoptotic effects against sepsis beyond its cardiovascular regulatory actions. However, the precise molecular targets and underlying mechanisms remain largely unclear [4–6]. Esmolol reduces HR and improves the balance between myocardial oxygen supply and demand [7–9]; Wang et al. reported that esmolol alleviates septic myocarditis by modulating the *α*7 nAChR/STAT3/NF‐*κ*B axis [10]; esmolol can also mitigate SAMI by regulating the AMPK/mTOR/ULK1 pathway and the TLR4/NF‐*κ*B signaling pathway [11, 12]. However, the key genes and molecular mechanisms underlying the cardioprotective effects of esmolol in SAMI remain unclear. The pathophysiological process of SAMI is highly complex, with apoptosis and inflammatory storms serving as the core driving mechanisms that interact closely with one another [13–15].

Apoptotic protease activating factor 1 (APAF1) is a pivotal hub molecule in the mitochondrial apoptotic pathway. It binds cytochrome C and dATP to form apoptosomes, interacts with caspase‐9, and triggers the caspase‐9/caspase‐3 cascade, thereby modulating apoptosis following myocardial injury. A study by Wang et al. demonstrated that specific APAF1 knockout markedly alleviated apoptosis induced by septic acute kidney injury in mice [16–18]. The JAK/STAT pathway is a promising therapeutic target for multiple cytokine‐mediated autoimmune and inflammatory diseases. During sepsis, stimuli such as LPS induce abnormal phosphorylation and activation of JAK2 and STAT3, which further promote excessive secretion of proinflammatory cytokines including IL‐1*β*, TNF‐*α*, and IL‐6, aggravating myocardial inflammation and tissue damage [19, 20]. An indirect regulatory interaction may exist between the JAK2/STAT3 pathway and APAF1. Under inflammatory conditions, activation of STAT3 and NF‐*κ*B signals occurs; upon exposure to TNF‐*α*, IKK*β* is phosphorylated and degraded, allowing NF‐*κ*B to translocate into the nucleus. Nuclear NF‐*κ*B not only stimulates the production of various inflammatory cytokines but also activates the APAF1/caspase‐9 axis, thereby driving apoptotic and inflammatory responses and exacerbating organ injury [18, 21]. Therefore, simultaneously targeting JAK2/STAT3 and APAF1 to block their cascade activation may represent a key strategy for the prevention and treatment of SAMI.

With the extensive application of bioinformatics in drug target identification, this study integrated bioinformatics analysis, molecular docking, an in vitro cardiomyocyte injury model, and a rat sepsis model to systematically investigate the protective effects of esmolol against SAMI to provide a novel therapeutic strategy for treating SAMI.

## 2. Materials and Methods

### 2.1. Preprocessing of Differentially Expressed Genes (DEGs) in SAMI

In this study, mRNA expression profiling data of heart tissues from SAMI and control mice were downloaded and analyzed from the GSE267388 dataset of the Gene Expression Omnibus (GEO) database (https://www.ncbi.nlm.nih.gov/geo/). The dataset included heart samples from five LPS‐induced septic mice and five control C57BL/6 male mice. Differential expression analyses were performed using the DESeq2 R package. Genes with |log2FoldChange| > 1 and *p* < 0.05 were identified as DEGs and used for subsequent analysis.

### 2.2. Prediction of Esmolol Target Proteins

The potential targets of esmolol were predicted using the PharmMapper web server (http://www.lilab-ecust.cn/pharmmapper/). The 3D structure file of esmolol was uploaded to the platform and 375 potential target proteins with high matching scores were obtained through pharmacophore model alignment. PharmMapper adopts a reverse screening strategy based on spatial pharmacophore features and ranks results using statistical methods.

### 2.3. Screening and Enrichment Analysis of Intersection Genes

DEGs and predicted esmolol targets were intersected to screen for common genes that may participate in the protective effects of esmolol against SAMI. Gene Ontology (GO) functional annotation and Kyoto Encyclopedia of Genes and Genomes (KEGG) pathway enrichment analyses were performed using the ClusterProfiler R package with a significance threshold of *p* < 0.05. The GO analyses included molecular function (MF), cellular components (CC), and biological processes (BP). KEGG analysis was applied to reveal potential regulatory pathways.

### 2.4. Molecular Docking

The three‐dimensional crystal structures of the proteins were obtained from the PDB database. Molecular docking analysis was performed using AutoDock Vina 1.2.5. Structural optimization of esmolol and protein pretreatment, including dehydration, hydrogenation, and Gasteiger charge calculations, was completed using AutoDockTools. A binding energy of less than −6.0 kcal/mol indicated strong binding stability. The docking results were visualized three‐dimensionally using Discovery Studio 2019 to analyze the hydrogen bonds, hydrophobic interactions, and key binding residues. The binding mode diagrams were generated using PyMOL 2.5.

### 2.5. Molecular Dynamics (MD) Simulation

To further characterize the binding stability and dynamic interaction features of esmolol with APAF1 and JAK2, all‐atom MD simulations were performed using the GROMACS 2024.5 software in conjunction with the CHARMM36 force field. The initial docking conformations of the APAF1–esmolol and JAK2–esmolol complexes were used as starting structures. Each complex was placed in a dodecahedral box filled with explicit TIP3P water molecules, with a minimum distance of 1.2 nm between the proteins and box boundary. Na^+^ or Cl^−^ ions were added to neutralize the net charge of the system. Energy minimization was performed using the steepest descent algorithm until the maximum intermolecular force converged to below 1000 kJ·mol^−1^·nm^−1^. Equilibrium simulations of 100 ps were performed on the system under NVT and NPT conditions, with the temperature maintained at 300 K using a v‐rescale thermostat, and the pressure maintained at 1 bar using a Parrinella–Rahman isobaric‐isothermal thermostat. A 100 ns production MD simulation was conducted under the same NPT conditions. All bond lengths were constrained using the LINCS algorithm and long‐range electrostatic interactions were calculated using the Particle‐Mesh Ewald (PME) method. The van der Waals interactions were truncated at 1.0 nm using a smooth truncation function. Trajectories were saved every 10 ps for subsequent analyses.

Trajectory analysis was performed using built‐in GROMACS tools, including root‐mean‐square deviation (RMSD) for the protein main chain and ligands, root‐mean‐square fluctuation (RMSF) for individual residues, radius of gyration (Rg), solvent‐accessible surface area (SASA), and number of intermolecular hydrogen bonds. Based on the first two principal components (PC1 and PC2) obtained from principal component analysis (PCA) of the protein main‐chain atoms, a two‐dimensional free energy landscape (FEL) was constructed, and the Gibbs free energy (*Δ*G) relative to the global minimum was calculated.

### 2.6. Cell Culture and Treatment

Rat H9C2 cardiomyocytes were purchased from Wuhan SUNNCELL Biotechnology Co. (SNL‐029). Cells were cultured in DMEM medium (SNM‐002A, SUNNCELL, China) supplemented with 10% fetal bovine serum (10099141, Gibco, United States) and 1% penicillin‐streptomycin solution (PB180120, Procell, China) in a cell incubator at 37°C with 5% CO_2_.

Cells were treated with 10 *μ*g/mL LPS (HY‐D1056, MCE, USA) for 24 h. After LPS treatment, some cells were incubated with esmolol hydrochloride injection (H20243036, Boshen Biopharmaceutical Co., Ltd., China) at concentrations of 0.1, 0.25, 0.5, 1, 2, and 4 *μ*M for another 24 h.

### 2.7. CCK‐8 Assay

H9C2 cells were seeded into 96‐well plates at a density of 5000 cells/well and cultured for 24 h, followed by treatment according to the experimental grouping. Ten microliters of CCK‐8 reagent (C0038, Beyotime, China) was added to each well, and the plates were incubated at 37°C in the dark for 2 h. The optical density (OD) of each well was measured at 450 nm. The relative cell viability of each group was calculated using the cell viability of the control group, which was set to 100%.

### 2.8. Flow Cytometry for Apoptosis Detection

H9C2 cells were seeded in 6‐well plates at a density of 1 × 10^6^ cells per well and cultured before treatment. Cells were digested with 0.25% EDTA–acid‐free trypsin (C0205; Beyotime, China) and collected. According to the instructions of the Annexin V‐FITC/PI Apoptosis Detection Kit (Cat. No. 556547, BD Biosciences, United States), cells were resuspended in 100 *μ*L of 1× Binding Buffer, incubated with 5 *μ*L of Annexin V‐FITC and 5 *μ*L of PI staining solution in the dark at room temperature for 15 min. Then, 400 *μ*L of 1× Binding Buffer was added. The cell apoptosis rate was detected using a flow cytometer (BD FACSCalibur, BD Biosciences, United States) and analyzed using FlowJo 10.8.1 software.

### 2.9. Measurement of Reactive Oxygen Species (ROS) Concentrations

Cells were incubated with 10‐*μ*M DCFH‐DA fluorescent probe (S0033S, Beyotime, China) at 37°C in the dark for 20 min. After washing with a serum‐free medium, the cells were digested, harvested, and resuspended in phosphate buffered saline (PBS). Intracellular fluorescence intensity was measured via flow cytometry at an excitation wavelength of 488 nm and an emission wavelength of 525 nm. The intracellularROS concentration was represented by the mean fluorescence intensity.

### 2.10. JC‐1 Assay

Cells were incubated with JC‐1 fluorescent probe (E‐CK‐A301, Elabscience, China) at 37°C in the dark for 20 min. After washing with the JC‐1 staining buffer, the cells were digested with trypsin, harvested, and resuspended. The fluorescence intensities of JC‐1 aggregates (excitation 525 nm, emission 590 nm) and JC‐1 monomers (excitation 488 nm, emission 530 nm) were detected using flow cytometry.

### 2.11. TUNEL Staining

H9C2 cells were seeded onto sterile coverslips in 24‐well plates at a density of 2 × 10^5^ cells per well and cultured for 24 h before group‐specific treatment. After incubation, the medium was discarded, and the cells were fixed with 4% paraformaldehyde (P1110, Solarbio, China) at room temperature for 30 min. After washing with PBS, the cells were incubated with 0.1% Triton X‐100 at room temperature for 5 min. Then, TUNEL reaction mixture (C1088, Beyotime, China) was added, followed by incubation at 37°C in the dark for 60 min. After three washes with PBS, the nuclei were counterstained with DAPI at room temperature for 5 min. After mounting, images were captured using a laser confocal microscope.

### 2.12. RT‐qPCR

Total RNA was extracted from the cells using TRIzol reagent (R1100, Solarbio, China). RNA was reverse transcribed into cDNA using a reverse transcription kit (K16225, Solarbio, China) according to the manufacturer′s instructions. Quantitative PCR amplification was performed using the SYBR Green PCR kit (SR1110, Solarbio, China) on a CFX96 Real‐Time PCR System (Bio‐Rad, United States). The reaction conditions were as follows: pre‐denaturation at 95°C for 30 s, followed by 40 cycles of denaturation at 95°C for 5 s and annealing at 60°C for 30 s. ACTB was used as the internal reference, and the relative gene expression was calculated using the 2^−*ΔΔ*Ct^ method. The primer sequences are listed in Table S1.

### 2.13. APAF1 Overexpression Plasmid Transfection

H9C2 cells were seeded into 6‐well plates at a density of 1 × 10^5^ cells per well and cultured until 70%–80% confluence. Transfection was performed using Lipofectamine 3000 (Cat. No. L3000015; Invitrogen) according to the manufacturer′s protocol. Briefly, the APAF1 overexpression plasmid and transfection reagent were diluted separately, incubated at room temperature for 15 min, and then added to the cell medium. The overexpression plasmid sequences are listed in Table S2. After 6 h, the medium was replaced with fresh complete medium, and cells were cultured for another 24 h. The transfection efficiency was verified before subsequent experiments.

### 2.14. Experimental Animals and Grouping

Thirty‐two SPF‐grade male Sprague‐Dawley rats weighing 200–220 g were purchased from Beijing Huafukang Biotechnology Co. Ltd. All animal experiments were approved by the Laboratory Animal Center of the Fourth Hospital of Hebei Medical University (license number: SYXK (Ji) 2022‐011; ethics number: IACUC‐4th Hos Hebmu‐20260006). Rats were housed in a barrier environment with a temperature of 22^°^C ± 2^°^C, humidity of 50%–60%, and a 12‐h light/dark cycle, with free access to food and water. After 1 week of adaptive feeding, the rats were randomly divided into four groups (*n* = 8 each) [22]: control, model, esmolol 25 mg/kg, and esmolol 50 mg/kg. The rats in the model and treatment groups received an intraperitoneal injection of LPS (10 mg/kg). After 6 h, emodolol (25 or 50 mg/kg) was administered intraperitoneally. Echocardiography was performed after anesthesia. Blood was then collected from the eyeball, and the rats were euthanized by an overdose of pentobarbital sodium via intraperitoneal injection. The heart tissues were harvested for subsequent experiments.

### 2.15. Echocardiography

Rats were anesthetized by intraperitoneal injection of pentobarbital sodium (50 mg/kg, P3761, Sigma‐Aldrich, United States), fixed on an operating table, and chest hair was removed with depilatory cream. Left ventricular M‐mode echocardiographic images were acquired using a small‐animal ultrasound system (Fujifilm Visual Sonics, Japan) with a 15 MHz probe. HR, left ventricular ejection fraction (EF%), left ventricular fractional shortening (FS%), left ventricular end‐diastolic diameter (LVEDD), and left ventricular end‐systolic diameter (LVESD) were calculated. To minimize observer bias, all echocardiographic measurements were performed by an experienced researcher blinded to group assignments.

### 2.16. Hematoxylin‐Eosin (HE) Staining

Rat myocardial tissues were fixed in 4% paraformaldehyde for 24 h, followed by routine dehydration, transparency, paraffin embedding, and sectioning into 4‐*μ*m continuous slices. After dewaxing with xylene and hydrating with gradient ethanol, the sections were stained with hematoxylin and eosin (SI101‐01, Solarbio, China). The sections were then dehydrated with a gradient of ethanol, cleared with xylene, and mounted with neutral balsam. Histopathological changes in myocardial tissues were observed and photographed under a light microscope (Model BX53, Olympus, Japan).

### 2.17. Detection of Oxidative Stress Markers

The cells were lysed in a cell lysis buffer (R0010, Solarbio, China) on ice for 30 min, and the supernatants were collected after centrifugation. The myocardial tissue (50 mg) was homogenized and centrifuged to obtain the supernatant. The concentrations or activities of MDA (S0131S, Beyotime, China), ATP, and SOD (ADS‐W‐A001‐96; ADS‐W‐KY011, AIDISHENG, China) were measured using the corresponding assay kits, following the manufacturer′s instructions.

### 2.18. Detection of Myocardial Injury Markers

Rat whole blood was incubated at room temperature for 30 min and centrifuged at 3000 rpm at 4°C for 15 min to separate serum. The concentrations of LDH (ADS‐W‐FM003‐96, AIDISHENG, China), CK‐MB, cTnI, and cTnT (JM‐02031R2; JM‐01833R1; JM‐10703R1, JingMei, China) were measured using commercial assay kits.

### 2.19. Measurement of Inflammatory Factors

The cell culture and tissue homogenate supernatants were collected. The concentrations of IL‐1*β*, TNF‐*α*, and IL‐6 were determined using rat ELISA kits (JL20884; JL13202; JL20896; JONLNBIO, China) according to the protocols. Absorbance was measured at 450 nm using a microplate reader, and cytokine concentrations were calculated using standard curves.

### 2.20. Western Blot

Cells or myocardial tissues were lysed on ice for 30 min and the supernatants were collected after centrifugation. The protein concentration was quantified using a BCA protein assay kit (P0010S, Beyotime, China). Equal amounts of proteins were separated by sodium dodecyl sulfate‐polyacrylamide gel electrophoresis and transferred to PVDF membranes. Membranes were blocked with 5% nonfat milk for 1 h at room temperature and incubated with primary antibodies overnight at 4°C. After washing with TBST, the membranes were incubated with horseradish peroxidase‐conjugated secondary antibodies for 2 h at room temperature. The details of the antibodies used are presented in Table S3. Protein bands were visualized using an ECL chemiluminescence kit (P0018AS, Beyotime, China), and band gray values were analyzed using ImageJ 1.53 software.

### 2.21. Cellular Thermal Shift Assay (CETSA)

Treat H9c2 cells with 2‐*μ*M esmolol or an equal volume of PBS (solvent control) for 24 h. After washing, the cells were detached by trypsin digestion and resuspended in a complete medium. Divide each suspension equally among eight PCR tubes and cycle them in a thermal cycler at 37°C, 47°C, 52°C, 55°C, 57°C, 61°C, 64°C, and 67°C, holding each temperature for 3 min. After heating, the cells were lysed by subjecting them to three consecutive freeze‐thaw cycles. Subsequently, the relative expression levels of soluble proteins in the lysates from each group were determined using western blot analysis.

### 2.22. Statistical Analysis

All experiments were performed independently at least thrice. Data are presented as mean ± standard deviation (SD). Statistical analyses were performed using GraphPad Prism software. Comparisons among multiple groups were performed using one‐way analysis of variance (ANOVA), followed by Tukey′s post hoc multiple comparisons for pairwise analysis if the overall difference was statistically significant. Statistical significance was set at *p* < 0.05 study.

## 3. Results

### 3.1. Screening of DEGs Associated With SAMI and Esmolol

The GSE267388 dataset and the potential target proteins of esmolol were downloaded and predicted. A total of 1429 SAMI‐related DEGs and 375 esmolol targets were identified using the R package. The intersection of DEGs and predicted esmolol targets yielded 51 common genes that may participate in the ameliorative effect of esmolol on SAMI (Figure 1A).

**Figure 1:**
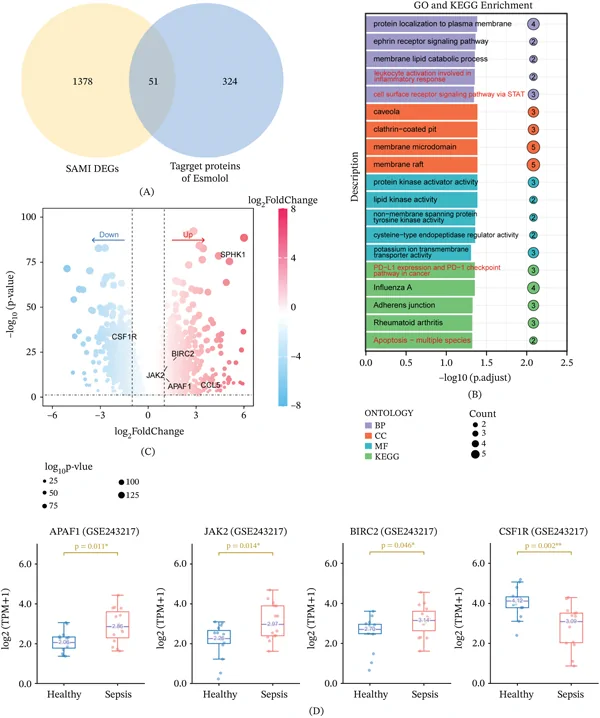
Screening and validation of core targets of esmolol in SAMI. (A) Venn diagram showing the overlap between SAMI‐related DEGs and esmolol target proteins. (B) GO/KEGG enrichment analysis of the 51 overlapping genes. (C) Volcano plot of SAMI‐related DEGs, highlighting core candidate genes. (D) The external dataset GSE243217 shows the expression of four targets with consistent trends. Data are presented as mean ± SEM.  ^∗^ *p* < 0.05,  ^∗∗^ *p* < 0.01.

### 3.2. Pathway Enrichment and Core Gene Screening

Subsequently, GO functional annotation and KEGG pathway enrichment analyses of the 51 overlapping genes were performed using ClusterProfiler R package, and significantly enriched GO terms and KEGG pathways are presented (Figure 1B). The results showed that the overlapping genes were mainly enriched in BP, including protein localization to the plasma membrane, the cell surface receptor signaling pathway via STAT, and leukocyte activation involved in the inflammatory response. For CC, genes are highly enriched in membrane microdomains and membrane rafts, which are functional subdomains of the cardiomyocyte membrane [23]. In terms of MF, protein kinase activator activity and potassium ion transmembrane transporter activity were enriched. KEGG pathway analysis indicated that the overlapping genes were enriched in the PD‐L1 expression and PD‐1 checkpoint pathways in cancer and apoptosis, suggesting that esmolol exerts cardioprotective effects in sepsis via a multidimensional molecular regulatory network [24].

Among the significantly enriched pathways, some were nonspecific (general) or associated with known diseases, such as protein localization to the plasma membrane, ephrin receptor signaling pathway, membrane lipid catabolic process, caveolae, clathrin‐coated pits, membrane microdomains, membrane rafts, influenza A, and rheumatoid arthritis. These pathways are not strongly associated with the disease under study, SAMI; in particular, terms related to focal adhesions and membranes describe general cellular structural features. Based on the enrichment results, four signaling pathways closely related to disease progression were selected for further investigation: PD‐L1 expression and PD‐1 checkpoint pathway in cancer (hsa05235) are central to sepsis‐induced immunosuppression and T‐cell dysfunction, both of which are hallmark features of SAMI; apoptosis—multiple species (hsa04215) mediates cardiomyocyte death and dysfunction in SAMI; cell surface receptor signaling pathway via STAT (GO:0097696) serves as the primary signaling axis linking proinflammatory cytokines to the transcriptional regulation of inflammation and apoptosis in cardiomyocytes; and leukocyte activation involved in inflammatory response (GO:0002269) drives excessive inflammation, cytokine release, and the infiltration of immune cells into the myocardium [15, 24–29]. These four pathways collectively reflect the core pathophysiological features of SAMI, including immune imbalance, cardiomyocyte apoptosis, and amplified inflammation. Apoptosis and inflammation are the most direct manifestations of sepsis‐induced organ damage. The treatment of SAMI with esmolol also primarily focuses on alleviating apoptosis and inflammation [30]; however, the literature on this topic is relatively scarce, and clearly identified specific targets are even rarer. Six significantly enriched core genes were extracted from these pathways: JAK2, SPHK1, CCL5, CSF1R, APAF1, and BIRC2, which are regarded as key candidate targets of esmolol (Figure 1C). To eliminate the selection bias that may arise from the analysis of a single transcriptomic dataset and enhance the robustness of the core target identification results, the human dataset GSE243217 was incorporated to perform an external validation of the expression profiles of the candidate core genes identified in the initial screening. The expression levels of APAF1, JAK2, BIRC2, and CSF1R were significantly higher in the sepsis group than in the healthy control group, which was consistent with the results from the initial analysis of dataset GSE267388, suggesting that the abnormal expression of these genes during the pathological process of sepsis exhibited good cross‐dataset stability. However, trends in the expression changes of SPHK1 and CCL5 differed between the two datasets, suggesting that their expression fluctuations may be influenced by dataset‐specific factors (Figure 1D).

### 3.3. Esmolol and APAF1 Can Bind Stably

The binding affinity of esmolol for these candidate targets was verified. The binding energies of esmolol to APAF1, SPHK1, BIRC2, JAK2, CCL5, and CSF1R were −7.73, −7.05, −6.30, −6.05, −4.46, and −4.27 kcal/mol, respectively. Thus, esmolol showed stable binding to APAF1, SPHK1, BIRC2, and JAK2 with the highest binding affinity for APAF1 (Figure 2A). We performed 100 ns full‐atom MD simulations of APAF1 and JAK2. FEL analysis revealed distinct low‐energy conformational basins in both complexes. Hydrogen bond analysis showed that esmolol maintained 1–3 persistent intermolecular hydrogen bonds with APAF1 and JAK2 throughout the simulations. Radius of gyration (Rg) analysis showed that the overall compactness of both proteins remained stable throughout the simulation, with the JAK2 complex stabilizing after a slight compression in the early stages. The protein backbone RMSD for both the APAF1‐esmolol and JAK2‐esmolol complexes increased rapidly within the first 20–30 ns of the simulation before leveling off, indicating that both systems reached structural equilibrium. The ligand RMSD remained low throughout the simulation (0.15–0.25 nm for the APAF1 complex and 0.10–0.25 nm for the JAK2 complex), suggesting that esmolol did not undergo significant dissociation or reorientation in either binding pocket. The RMSF analysis indicated that the residues in the binding pockets of both proteins exhibited low flexibility. In summary, the MD simulation results indicated that esmolol forms stable and persistent complexes with both APAF1 and JAK2, providing kinetic and thermodynamic support for their direct physical binding (Figures S1 and S2).

**Figure 2:**
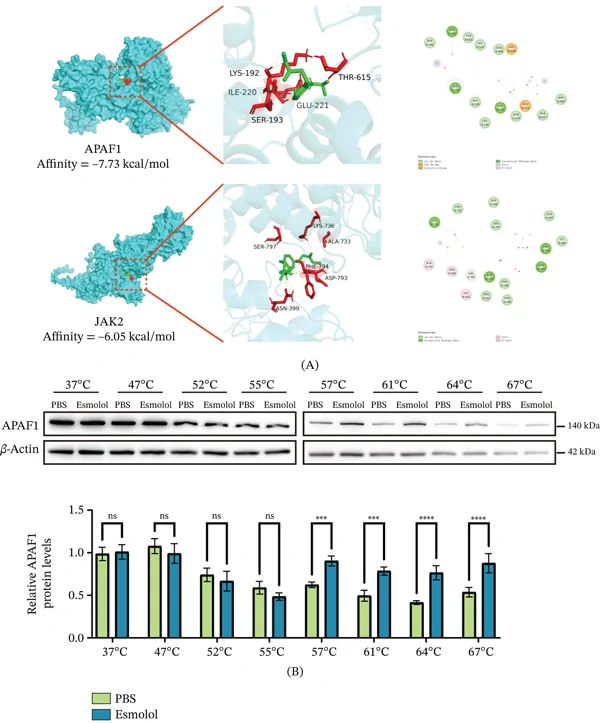
Molecular docking of esmolol with key targets and CETSA validation. (A) Molecular docking of esmolol with APAF1 and JAK2 (binding affinities: −7.73 and −6.05 kcal/mol, respectively). The interaction bonds are shown on the right; (B) CETSA validation of the direct binding of esmolol to APAF1 in cells. Data are presented as mean ± SEM. ns, not significant;  ^∗∗∗^ *p* < 0.001,  ^∗∗∗∗^ *p* < 0.0001.

To validate the molecular docking predictions and confirm that esmolol directly bound APAF1, we performed a CETSA assay. As shown in Figure 2B, no statistically significant difference in APAF1 protein concentrations was detected between the esmolol‐treated group and the PBS control group at temperatures ranging from 37°C to 55°C. However, upon exposure to higher temperatures (57°C, 61°C, 64°C, and 67°C), APAF1 protein concentrations in the esmolol‐treated group were significantly higher (*p* < 0.001), demonstrating that esmolol physically interacts with APAF1 within the cells. Given that APAF1 is upregulated in SAMI, and in combination with pathway enrichment, we hypothesized that esmolol may attenuate apoptosis by directly inhibiting APAF1 activity, thereby protecting cardiomyocyte function.

### 3.4. Esmolol Ameliorates LPS‐Induced Reduction in H9C2 Cell Viability

Kavakcıoğlu Yardımcı et al. previously treated lung cells with different *β*‐blockers and found that low‐dose esmolol exerted no effect on lung cell viability [31]. Accordingly, we evaluated the protective effects of esmolol against LPS‐induced cardiomyocyte injury. Compared with the control group, treatment with 10 *μ*g/mL LPS for 24 h significantly reduced the relative viability of H9C2 cells. Previous studies have shown that esmolol can produce distinct biological effects even at low molar concentrations (≥ 1 *μ*M) [32]; therefore, we chose to pretreat the cells with concentrations ranging from 0.1 to 4 *μ*M. Cell viability was increased as the concentration of esmolol increased from 0.1 to 2 *μ*M. Notably, 2‐*μ*M esmolol produced the most pronounced protective effect, with cell viability restored to 83.03%. No further improvement in cell viability was observed at 4‐*μ*M esmolol (Figure 3A). This may be due to the action of *β*1‐adrenergic receptors and their targets. Further increase in concentration did not produce additional receptor blockade; in contrast, even higher concentrations may induce cytotoxicity. Furthermore, because the total number of intracellular targets is limited, further increases in drug concentration do not lead to additional inhibition of target function. These are typical pharmacological phenomena that explain why no further improvement was observed at 4 *μ*M. Additionally, owing to cost reasons, no treatment groups with higher doses were included. Therefore, 1 and 2 *μ*M were chosen as the working concentrations of esmolol in subsequent mechanistic experiments.

**Figure 3:**
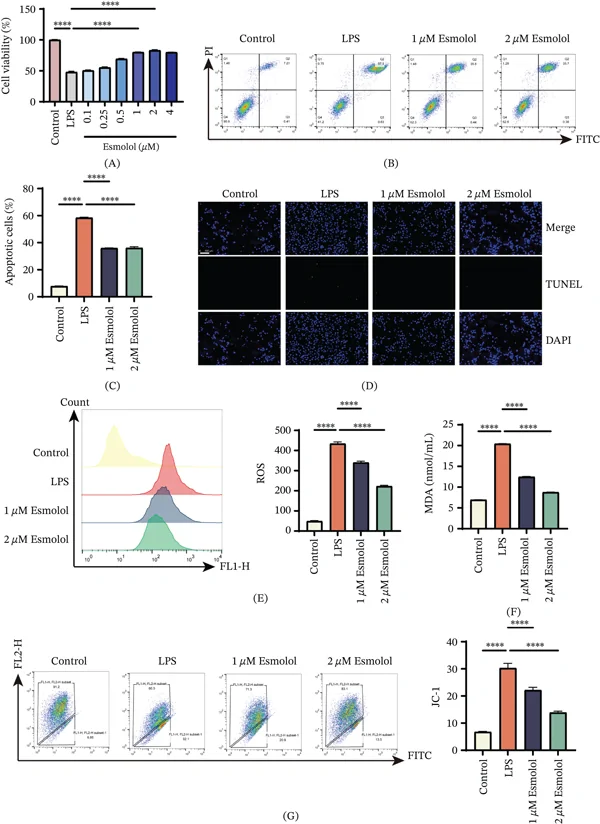
Esmolol alleviates LPS‐induced injury, apoptosis, and oxidative stress in H9C2 cardiomyocytes. (A) CCK‐8 assay for cell viability (*n* = 6); (B–C) Flow cytometry analysis of apoptosis rates; (D) representative image of TUNEL staining; (E) flow cytometry analysis of intracellular ROS levels; (F) kit‐based assay for MDA levels; (G) flow cytometry profile of JC‐1 staining (*n* = 3). Data are presented as mean ± SEM.  ^∗∗∗∗^ *p* < 0.0001.

### 3.5. Esmolol Significantly Alleviates LPS‐Induced Apoptosis and Oxidative Damage in H9C2 Cells

Apoptosis is the core pathological process in SAMI. The apoptotic rate was only 7.82% in the control group, but was markedly elevated in the LPS model group, and was significantly reduced by 1 and 2‐*μ*M esmolol treatment (*p* < 0.0001, Figure 3B,C). The results of TUNEL staining were consistent with those of flow cytometry; few positive cells were observed in the control group, whereas abundant TUNEL‐positive cells were observed in the LPS group. Esmolol treatment notably decreased the number of positive cells, with a better effect at 2 *μ*M, further confirming that esmolol markedly suppresses cardiomyocyte apoptosis (Figure 3D).

Oxidative stress is a crucial upstream pathway that mediates apoptosis. Excessive ROS directly damages biological macromolecules and triggers apoptotic signaling [33]. Intracellular ROS concentrations and MDA content significantly increased in the LPS model group. Esmolol reduced the ROS concentrations and MDA production, effectively alleviating oxidative stress (Figure 3E,F). Mitochondria are the main sources of ROS. LPS stimulation caused a marked decrease in mitochondrial membrane potential, which was significantly ameliorated by esmolol treatment (Figure 3G).

### 3.6. Esmolol Downregulates APAF1 Expression and Inhibits the Activation of the Mitochondrial Apoptotic Pathway

These findings indicated that esmolol inhibited cardiomyocyte apoptosis by mitigating oxidative stress and maintaining mitochondrial function. APAF1 is a core component of apoptosomes. Ferraro et al. demonstrated that APAF1‐deficient cells undergo metabolic reprogramming and exhibit enhanced resistance to apoptosis [34]. Therefore, in this study, we determined the expression levels of APAF1 and its downstream apoptosis‐related proteins. RT‐qPCR revealed that the mRNA level of APAF1 was markedly upregulated in the LPS model group, whereas esmolol treatment downregulated APAF1 transcription. Western blot analysis further verified the significant elevation in APAF1 protein expression in LPS‐treated H9C2 cells. Meanwhile, the concentrations of proapoptotic proteins (Bax, cleaved‐caspase‐3, cleaved‐caspase‐9) were increased, whereas the antiapoptotic protein Bcl‐2 was downregulated. However, esmolol treatment effectively reversed these effects. Collectively, esmolol may inhibit APAF1 expression, thereby blocking the activation of the mitochondrial apoptotic cascade and exerting antiapoptotic effects on cardiomyocytes (Figure 4A).

**Figure 4:**
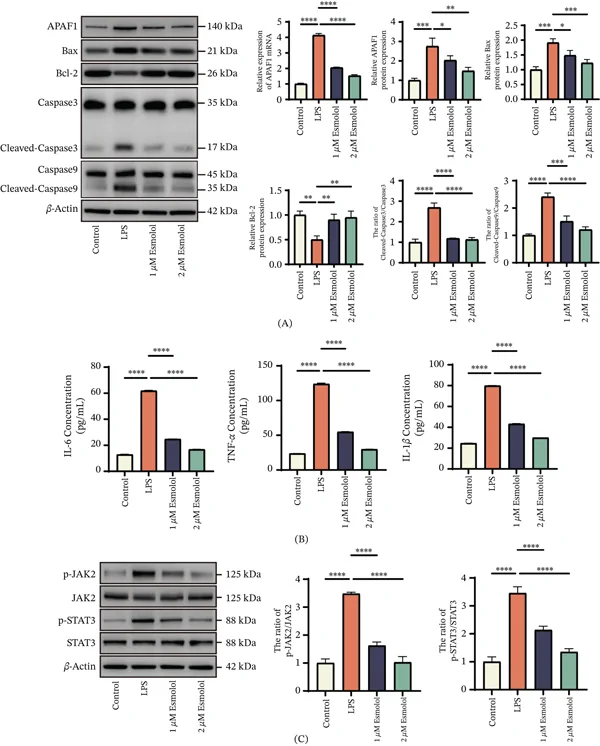
Esmolol inhibits LPS‐induced apoptosis in H9C2 cells and the activation of the JAK2/STAT3 inflammatory pathway (A) Western blot analysis of APAF1 and the apoptosis pathway; (B) ELISA analysis of proinflammatory cytokines; (C) Western blot analysis of the JAK2/STAT3 pathway (n = 3). Data are presented as mean ± SEM.  ^∗^ *p* < 0.05,  ^∗∗^ *p* < 0.01,  ^∗∗∗^ *p* < 0.001,  ^∗∗∗∗^ *p* < 0.0001.

### 3.7. Esmolol Inhibits the JAK2/STAT3 Signaling Pathway and Reduces the Secretion of Inflammatory Factors

As shown in Figure 4B, the concentrations of IL‐1*β*, TNF‐*α* and IL‐6 in the cell culture supernatant were significantly higher in the LPS model group than in the control group, whereas esmolol intervention reduced the secretion of these inflammatory factors. In Figure 4C, the ratios of p‐JAK2/JAK2 and p‐STAT3/STAT3 were markedly elevated in the LPS group, and these increases were suppressed by esmolol treatment. Taken together, these results indicate that esmolol alleviates LPS‐induced inflammatory responses in cardiomyocytes by inhibiting the activation of the JAK2/STAT3 signaling pathway.

### 3.8. Overexpression of APAF1 Reverses the Protective Effect of Esmolol on LPS‐Induced H9C2 Cell Injury

These experiments confirmed that esmolol alleviated apoptosis and related phenotypic changes, possibly by acting on APAF1. Subsequently, APAF1 was overexpressed in the H9C2 cells. The mRNA expression of APAF1 was significantly increased in the LPS + oe‐APAF1 group, indicating the successful construction of the APAF1 overexpression model (Figure 5A). After esmolol treatment, APAF1 mRNA concentrations were markedly downregulated in the LPS + Esmolol group compared with those in the LPS group. Moreover, 2‐*μ*M esmolol effectively reversed the upregulation of APAF1 mRNA induced by LPS and APAF1 overexpression in the LPS + oe‐APAF1 group (Figure 5B). Western blotting further verified the protein expression pattern of APAF1, which was consistent with the trend observed in the mRNA expression results (Figure 5C).

Figure 5Overexpression of APAF1 reverses the protective effect of esmolol against LPS‐induced damage in H9C2 cells (A) mRNA expression levels of APAF1 in H9C2 cells; (B) changes in APAF1 mRNA expression induced by LPS and APAF1 overexpression; (C) protein expression levels and quantitative analysis of APAF1 in H9C2 cells; (D) cell viability assay using the CCK‐8 method (*n* = 6); (E) Apoptosis rate assessed by flow cytometry. (F) Representative images of TUNEL staining; (G) flow cytometry analysis of intracellular ROS levels; (H) MDA levels (*n* = 3). Data are presented as mean ± SEM.  ^∗^ *p* < 0.05,  ^∗∗∗^ *p* < 0.001,  ^∗∗∗∗^ *p* < 0.0001.

**Figure:**
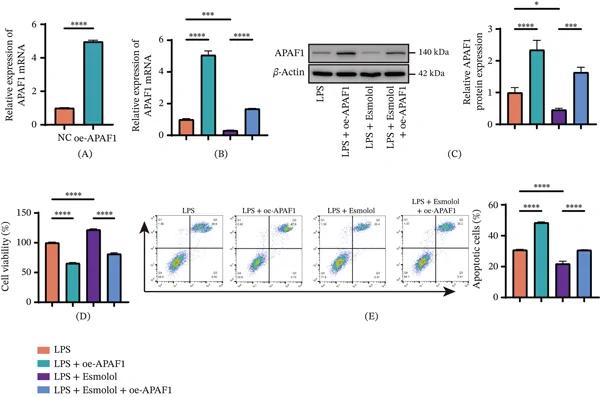

**Figure:**
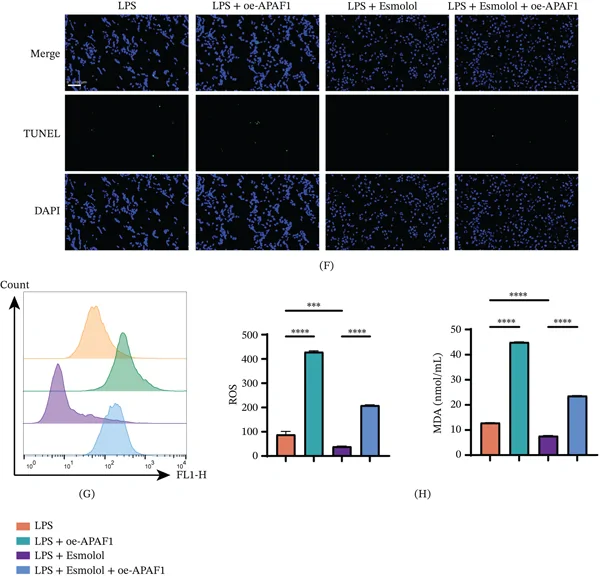

To clarify the role of APAF1 in the cardioprotective effect of esmolol, the CCK‐8 assay, flow cytometry, TUNEL staining, and oxidative stress indicator detection were performed. CCK‐8 results showed that compared with the LPS group, cell viability was further decreased in the LPS + oe‐APAF1 group, aggravating cellular injury, whereas cell viability was notably rescued in the LPS + Esmolol group. Notably, esmolol treatment restored cell viability in the LPS + oe‐APAF1 group, suggesting that esmolol could reverse cell damage caused by APAF1 overexpression (Figure 5D). Flow cytometry and TUNEL staining yielded results consistent with the CCK‐8 assay, further demonstrating that APAF1 mediates the antiapoptotic effects of esmolol (Figure 5E,F). Meanwhile, ROS and MDA concentrations were significantly elevated in the LPS + oe‐APAF1 group and reduced after esmolol intervention. Collectively, these findings indicated that esmolol exerts myocardial protection by downregulating APAF1, and APAF1 serves as a key molecular target of esmolol (Figure 5G,H).

In addition, we examined the activation levels of the JAK2/STAT3 pathway in each group and found that APAF1 overexpression, with or without esmolol treatment, did not affect the phosphorylation levels of JAK2 and STAT3. This provides preliminary support for the description of the JAK2/STAT3‐APAF1 axis in the existing literature; however, the specific mechanism by which JAK2/STAT3 regulates APAF1 requires further experimental investigation (Figure S3).

### 3.9. Esmolol Improves Myocardial Injury and Oxidative Stress in Septic Rats

Cardiac dysfunction is the most prominent clinical manifestation of SAMI and directly affects patient prognosis. Echocardiographic examination revealed that compared with the control group, rats in the LPS model group exhibited an increased HR, significantly decreased EF% and FS%, and markedly enlarged LVEDD and LVESD, indicating severe impairment of left ventricular systolic and diastolic function. Treatment with 25 and 50 mg/kg esmolol improved cardiac function indicators (Figure 6A). HE staining revealed regular myocardial structure and neatly arranged cardiomyocytes in the control group. In contrast, the model group displayed a disordered cellular arrangement, obvious cellular degeneration, and myocardial fiber rupture, whereas esmolol intervention markedly alleviated the histopathological damage (Figure 6B). Moreover, the myocardial MDA content was significantly increased, accompanied by decreased ATP content and SOD activity in the model group. Esmolol reversed these abnormalities in oxidative stress and energy metabolism, with the greatest effect observed at 50 mg/kg (Figure 6C). In addition, serum concentrations of LDH, CK‐MB, cTnI and cTnT, as well as myocardial TNF‐*α*, IL‐1*β* and IL‐6, were remarkably elevated in the model group, indicating severe myocardial necrosis and inflammatory response. The concentrations of these indicators were reduced following esmolol treatment (Figure 6D,E). Collectively, esmolol ameliorates cardiac dysfunction and relieves oxidative stress and inflammatory responses, thereby exerting protective effects against SAMI.

**Figure 6:**
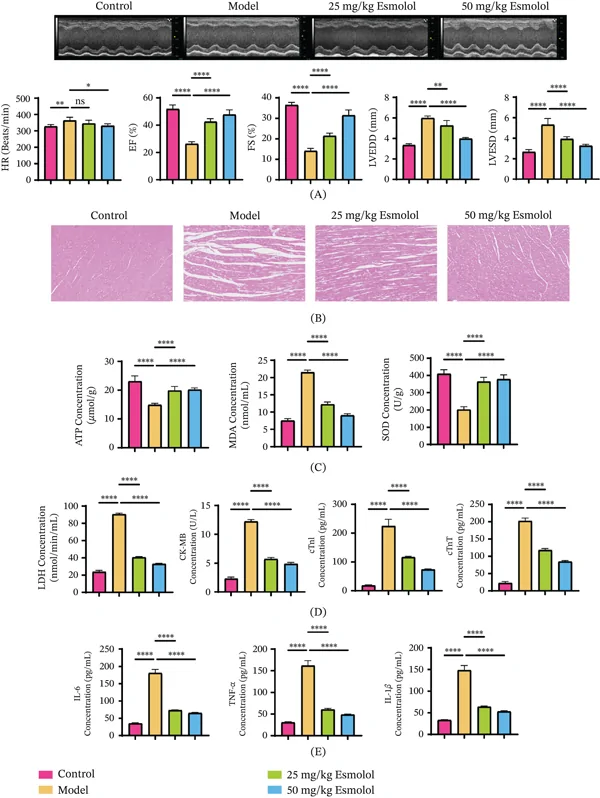
Esmolol improves cardiac function, myocardial pathological damage, oxidative stress, and inflammatory responses in rats with sepsis. (A) Representative images of rat echocardiography and quantitative analysis of cardiac function parameters (HR, EF, FS, LVEDD, LVESD) (*n* = 3); (B) representative images of HE‐stained rat myocardial tissue (*n* = 3); (C) detection of ATP content, MDA content, and SOD activity in rat myocardial tissue (*n* = 6); (D) detection of myocardial injury markers (LDH, CK‐MB, cTnI, cTnT) in rats (*n* = 6); (E) detection of myocardial inflammatory factors (IL‐6, TNF‐*α*, IL‐1*β*) in rats (*n* = 6). Data are presented as mean ± SEM. ns, not significant;  ^∗^ *p* < 0.05,  ^∗∗^ *p* < 0.01,  ^∗∗∗∗^ *p* < 0.0001.

### 3.10. Esmolol Exerts Cardioprotective Effects by Regulating the APAF1‐Mediated Apoptotic Pathway and JAK2/STAT3 Inflammatory Pathway

APAF1 is a key molecule in the apoptotic signaling pathway and has been preliminarily identified as an effective target of esmolol through bioinformatics and cellular experiments. Compared with the control group, the protein concentrations of APAF1 and proapoptotic molecules (Bax, cleaved‐caspase‐3, and cleaved‐caspase‐9) were significantly upregulated, whereas the antiapoptotic protein Bcl‐2 was downregulated in the myocardial tissues of septic rats. Esmolol intervention reversed these abnormal protein expression levels and notably suppressed the expression of APAF1 and downstream proapoptotic proteins (Figure 7A).

**Figure 7:**
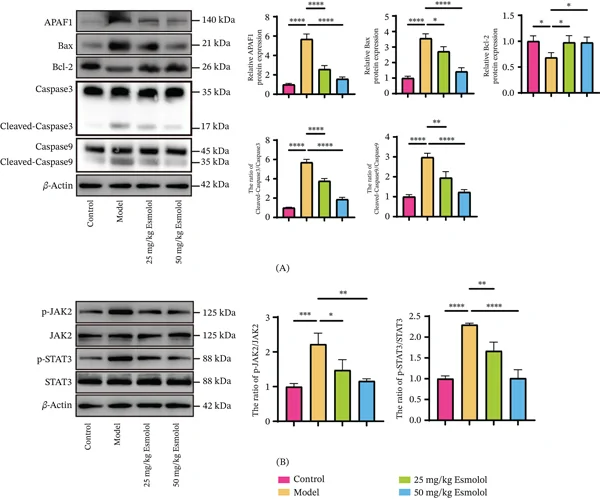
Esmolol exerts cardioprotective effects by regulating the APAF1 apoptotic pathway and the JAK2/STAT3 inflammatory pathway. (A) Expression of APAF1 and apoptosis‐related proteins in rat myocardial tissue (*n* = 3); (B) detection of JAK2/STAT3 phosphorylation levels in rat myocardial tissue (*n* = 3). Data are presented as mean ± SEM.  ^∗^ *p* < 0.05,  ^∗∗^ *p* < 0.01,  ^∗∗∗^ *p* < 0.001,  ^∗∗∗∗^ *p* < 0.0001.

Furthermore, we explored the central role of the JAK2/STAT3 pathway in regulating inflammatory responses during sepsis. The phosphorylation levels of JAK2 and STAT3 markedly increased in the model group, whereas esmolol treatment inhibited JAK2 and STAT3 activation (Figure 7B). These results suggest that esmolol alleviates the pathological progression of SAMI by suppressing the activation of the APAF1‐dependent apoptotic pathway and JAK2/STAT3 inflammatory pathways.

## 4. Discussion

Sepsis is a systemic inflammatory response triggered by bacterial infections or other pathogenic factors that can lead to multiple organ dysfunction. SAMI is a leading cause of mortality in patients with sepsis, and its pathological mechanisms involve excessive inflammatory responses, mitochondrial dysfunction, and cardiomyocyte apoptosis [35]. As a short‐acting *β*1‐adrenergic receptor blocker commonly used in clinical practice, this study identified esmolol as a functional effector molecule with APAF1 at its core. Esmolol exerts a protective effect against SAMI by inhibiting activation of the APAF1‐mediated apoptotic pathway and JAK2/STAT3 inflammatory signaling cascade.

Core targets shared by SAMI and esmolol, such as JAK2, SPHK1, CCL5, CSF1R, APAF1, and BIRC2, were screened using bioinformatic analyses. Molecular docking showed that the binding energies of esmolol to these targets were all lower than −4.0 kcal/mol, among which APAF1 exhibited the highest binding affinity. These six targets provide a roadmap for future functional studies (such as gene knockout/overexpression experiments) and constitute a targeted, biologically plausible list of candidates that can guide subsequent research. SAMI is characterized by exaggerated inflammation and massive cardiomyocyte apoptosis. APAF1 acts as an essential component of the apoptosome. During mitochondrial apoptosis, activated APAF1 initiates the caspase cascade and ultimately promotes cell death [16, 17]. Previous studies demonstrated that APAF1 participates in multiple cell death processes, including p53‐induced apoptosis and neuronal apoptosis [36]. Aberrant APAF1 expression is closely correlated with sepsis‐related organ injury, and targeted inhibition of APAF1 effectively alleviates sepsis‐induced lung injury, acute kidney injury, and myocardial damage [16, 18, 37].

Clinically, esmolol reduces the mortality, HR, and systemic inflammation in patients with sepsis [10, 30]. In addition, esmolol attenuates cardiomyocyte apoptosis, inflammatory response, and cardiac dysfunction in septic rats, providing solid theoretical support for our in vivo experimental design [38, 39]. Nevertheless, the direct molecular targets and precise signaling mechanisms of esmolol in SAMI remain unclear. To date, Wang et al. have reported that esmolol alleviates septic myocarditis by modulating the *α*7 nAChR/STAT3/NF‐*κ*B axis [10]. Zhang et al. identified Akt1 and mTOR as key genes and highlighted the PI3K/Akt signaling pathway as a candidate pathway associated with sepsis‐induced myocardial injury and response to esmolol [40]. Esmolol can also alleviate SAMI by regulating the AMPK/mTOR/ULK1 pathway and the TLR4/NF‐*κ*B signaling pathway [11, 12]. Based on this, and through bioinformatics predictions and experimental validation, this study proposes a new regulatory mechanism: in the context of SAMI, esmolol blocks mitochondrial apoptosis by directly acting on APAF1.

To further confirm the core interaction between esmolol and APAF1, cell experiments showed that esmolol reversed LPS‐induced damage in H9C2 cardiomyocytes. The effect gradually increased in the range of 0.1–2 *μ*M. Both TUNEL staining and flow cytometry confirmed that esmolol significantly reduced the rate of cardiomyocyte apoptosis, which is consistent with previous findings that *β*‐adrenergic receptor blockers inhibit programmed myocardial cell death. At the mechanistic level, LPS stimulation significantly upregulated the expression of APAF1 and its downstream molecules (Bax, cleaved‐caspase3/9), and esmolol reversed this trend, confirming that esmolol exerts antiapoptotic effects by inhibiting the APAF1‐mediated apoptotic pathway. Meanwhile, we found that esmolol significantly inhibited the phosphorylation and activation of JAK2 and STAT3, reduced the secretion of proinflammatory cytokines such as IL‐1*β*, TNF‐*α*, and IL‐6, and alleviated inflammatory infiltration in the myocardium. The JAK2/STAT3 pathway is one of the most extensively studied inflammatory signaling axes in sepsis. As described in Section 1, abnormal activation of the JAK2/STAT3 pathway may induce NF‐*κ*B nuclear translocation, which in turn activates the APAF1/caspase‐9 axis, thereby driving apoptosis and inflammation and exacerbating organ damage [18, 21]. We observed that the phosphorylation levels of JAK2/STAT3 were not significantly affected by APAF1 overexpression. Therefore, it is possible that esmolol also influences APAF1 expression via the JAK2/STAT3 pathway. However, the approach requires extensive experimental validation. For example, using JAK2/STAT3 inhibitors or siRNA in cells, as well as conducting more in‐depth APAF1 promoter reporter and ChIP analyses, could help investigate how the JAK2/STAT3 pathway influences APAF1 expression.

In vivo experiments further confirmed the conclusions obtained from the in vitro studies. Echocardiography results showed that esmolol effectively improved left ventricular systolic and diastolic function and alleviated histopathological damage, including disordered myocardial arrangement and myocardial fiber rupture. Meanwhile, esmolol decreased the serum levels of the myocardial injury markers LDH, CK‐MB, cTnI, and cTnT and suppressed oxidative stress as well as the release of proinflammatory cytokines in myocardial tissues. Moreover, APAF1 expression was gradually downregulated, accompanied by the suppression of apoptotic and inflammatory signaling pathways, which was more pronounced at 50 mg/kg. These trends are consistent with previous reports in a rat model of septic cardiomyopathy treated with esmolol [10]. Wang et al. also observed the antiapoptotic properties of esmolol in an LPS‐induced C57BL/6 mouse model [4]. Such animal models provide reliable support for understanding the molecular mechanisms underlying SAMI.

This study has certain limitations. Regarding the inhibition of the JAK2/STAT3 pathway, this study did not explore the upstream‐downstream temporal relationship between its regulation and APAF1, nor did it clarify whether there is an independent regulatory mechanism between them. In addition, the direct regulatory effect of esmolol on HR and hemodynamics in the sepsis model may contribute to the improvement of cardiac function. Although in vitro experiments using H9C2 cells excluded the interference of hemodynamics, the complexity of the in vivo environment requires further experiments to distinguish the specific contribution of each factor. The model system used in this study (i. p. administration of LPS plus a single esmolol injection) also had limitations. Future studies should be conducted using the CLP model as well as pharmacokinetic/pharmacodynamic (PK/PD) studies to more accurately correlate preclinical doses with clinical exposure levels.

In summary, this study confirmed that esmolol effectively alleviated sepsis‐induced myocardial injury by inhibiting APAF1‐mediated apoptosis and JAK2/STAT3‐mediated inflammation. Thus, APAF1 may be an effective target through which esmolol exerts its protective effects, thus providing new experimental evidence for the optimization of clinical treatment strategies for SAMI.

## Author Contributions

**Lei Zhao:** conceptualization, data curation, software, and writing—original draft. **Hua Dong:** validation, supervision, and methodology. **Shimin Dong:** formal analysis, writing—review and editing.

## Funding

No funding was received for this manuscript.

## Ethics Statement

All animal experiments in this study were conducted in accordance with the guidelines for the care and use of laboratory animals and were approved by the Experimental Animal Welfare Ethics Committee of the Fourth Hospital of Hebei Medical University (Approval No. IACUC‐4th Hos Hebmu‐20260006).

## Consent

The authors have nothing to report.

## Conflicts of Interest

The authors declare no conflicts of interest.

## Supporting Information

Additional supporting information can be found online in the Supporting Information section.

## Supporting information


**Supporting Information 1** Figure S1: Molecular dynamics simulation analysis of the APAF1–esmolol complex over 100 ns. Figure S2: Molecular dynamics simulation analysis of the JAK2–esmolol complex over 100 ns. Figure S3: Effect of APAF1 overexpression on phosphorylation levels in the JAK2/STAT3 pathway.

**Supporting Information 2** Table S1: Oligonucleotide primers for qRT‐PCR. Table S2: Overexpression plasmid sequences. Table S3: Antibody information used in this study.

## Data Availability

The data that support the findings of this study are available from the corresponding author upon reasonable request.
