# Supplementary material for: Esmolol Alleviates Sepsis‐Associated Myocardial Injury by Acting on the APAF1 and JAK2/STAT3 Inflammatory Pathways
Source: Cardiovasc Ther. 2026 Oct 12;2026:2843754. doi: 10.1155/cdr/2843754 (PMC13656344; doi:10.1155/cdr/2843754)
Supplement: Supplementary file 1 [file CDR-2026-2843754-s001.docx]

**Figure S1. Molecular dynamics simulation analysis of the APAF1–esmolol complex over 100 ns.**

**Figure S2. Molecular dynamics simulation analysis of the JAK2–esmolol complex over 100 ns.**

**Figure S3. Effect of APAF1 Overexpression on Phosphorylation Levels in the JAK2/STAT3 Pathway.**
